# Intelligent Plasmonic Bio‐Holoelectrochemical Systems: A Testable Architecture for Light‐Programmed Microbial Electrochemistry

**DOI:** 10.1002/gch2.70160

**Published:** 2026-09-15

**Authors:** Timoth Mkilima

**Affiliations:** ^1^ Department of Environmental Engineering and Management The University of Dodoma Dodoma Tanzania

**Keywords:** bioelectrochemical systems, digital twins, microbial electrosynthesis, model predictive control, plasmonic interfaces, structured light

## Abstract

Microbial electrochemical systems couple extracellular electron transfer with substrate oxidation or CO_2_ reduction, but their performance is constrained by biofilm heterogeneity, transport gradients, electrode polarization, and community drift. This review examines intelligent plasmonic bio‐holoelectrochemical systems (iPBHS) as an evidence‐derived reference architecture integrating transparent bioelectrochemical reactors, optically addressable electrodes, structured‐light delivery, spatial sensing, and model‐predictive control. The synthesis focuses on four themes: plasmonic modulation of biomass‐normalized electron transfer, spatial transfer of projected photon fields into electrochemical responses, feedback control under safety and conservation constraints, and electron‐product carbon routing. Particular attention is given to mechanistic discrimination using resonant and off‐resonant illumination, temperature‐equivalent and structurally matched controls, spatially resolved measurements, and factorial interaction analysis. Evidence from microbial electrosynthesis, plasmonic photoelectrochemistry, optogenetic biofilms, digital twins, and reactor‐scale engineering is critically compared, while incompatible component‐level gains are not pooled. Carbon, electron, energy, and life‐cycle accounting provide the basis for evaluating system performance and scalability. No complete iPBHS prototype has yet been demonstrated, making cumulative validation of its constituent mechanisms the central research priority.

## Graphical abstract

An evidence‐derived architecture integrates structured light, plasmonic bioelectrodes, spatial sensing, microbial electrosynthesis, and digital‐twin control into a testable framework for light‐programmed microbial electrochemistry. Mechanistic discrimination, equal‐dose spatial validation, and conservation‐aware performance accounting define the conditions required to separate genuine optical‐electrochemical coupling from thermal, structural, and control artefacts before credible scale‐up.

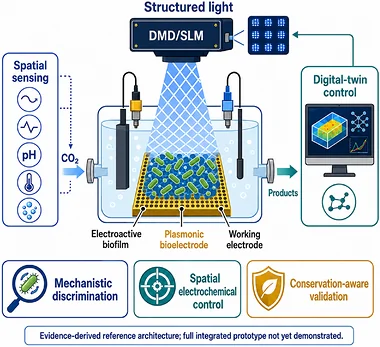

## 1 Introduction

Bioelectrochemical and photoelectrochemical systems provide complementary control over reaction selectivity and photon‐driven charge generation [1, 2]. Microbial fuel cells and microbial electrosynthesis exploit biologically selective redox pathways, but current density and product distribution remain functions of biofilm architecture, extracellular electron‐transfer route, substrate transport, and community dynamics [3, 4, 5]. Plasmonic and semiconductor photoelectrodes enhance light absorption and charge separation, although most mechanistic evidence derives from abiotic interfaces with tightly controlled catalyst and electrolyte properties [2, 6, 7]. Integration of these domains therefore depends on co‐registration of the photon field, electrode polarization, transport state, and microbial response rather than inference from bulk current or product concentration.

Plasmonic photoelectrochemistry provides a mechanistic basis for wavelength‐selective interfacial actuation [2, 6, 7]. Gold‐modified TiO_2_ nanotubes enhance visible‐light photoelectrochemical response, and multicomponent Z‐scheme systems improve charge separation and solar utilization [6, 7]. Photoactivated nanobiosensors further demonstrate red‐ and near‐infrared‐dependent signal transduction under conditions that limit direct photodamage [8]. These systems establish wavelength‐dependent optical transduction but do not resolve extracellular electron‐transfer kinetics or carbon flux in a living bioelectrode. In biological reactors, apparent illumination responses can be confounded by changes in conductivity, roughness [9, 10], local temperature [11, 12], photocatalysis [11], mass transport [12], or viability; orthogonal perturbations are therefore required for mechanistic discrimination [11, 12].

Light responsiveness is also established within microbial systems, although its functional meaning depends on organism and experimental scale. Natural microbial mats contain phototrophic, photoheterotrophic, and photoprotective guilds that redistribute carbon under illumination [13], while isotope tracing shows that light can change particulate organic‐matter utilization by attached and free‐living marine bacteria [14]. Microbe‐mineral interactions can further modify photochemical transformation of organic matter [15]. Collectively, these studies establish biological responsiveness to optical and mineral context, but not co‐registered optical actuation and electrochemical feedback at reactor scale.

Spatial biofilm engineering provides the closest experimental precedent for optically addressable electrode‐associated biomass. Light‐induced genetic circuits have patterned conductive Shewanella oneidensis biofilms on transparent electrodes and related pattern dimensions to electrical conduction [16]. Multiwavelength optogenetic lithography has generated spatially organized multi‐strain biofilms at millimeter resolution [17]. Broader developments in light‐responsive microbial systems and AI‐enabled bioelectrochemical analysis extend optical sensing and actuation capabilities [18, 19]. Collectively, these studies resolve light‐defined biofilm architecture but not dynamic control of local overpotential or online carbon flux. The unresolved integration variable is simultaneous registration of photon dose, electrochemical field, and biological state.

Cathodic microbial electrosynthesis provides the most direct carbon‐conversion reference because electrode‐derived electrons support CO_2_ reduction to acetate and other extracellular organic products in Sporomusa ovata biofilms [3]. This capability has been demonstrated across several acetogenic species, including Sporomusa sphaeroides, Clostridium ljungdahlii, and Moorella thermoacetica, with organism‐dependent electron recovery, product composition, and electrosynthetic performance [4]. Light‐induced patterning of electroactive Shewanella oneidensis biofilms on transparent electrodes provides a complementary anodic reference in which illumination controls biofilm geometry and electrical conduction [16]. The cathodic configuration resolves carbon conversion, coulombic efficiency, and product selectivity, whereas the anodic configuration resolves spatial EET and interfacial conductivity. Quantum‐coherence measurements [20, 21], life‐cycle greenhouse‐gas performance [22], multi‐contaminant transformation [23], and distributed coordination [24, 25] introduce additional state variables and accounting boundaries beyond the reference reactor. Recent reactor studies show that electrodialysis‐assisted product removal [26], electrolytic gas‐lift operation [27], high‐rate plate‐reactor design [28], and intermittent‐power operation [29] can materially alter acetate productivity, product titer, and recovery dynamics without changing the core acetogenic chemistry.

This review evaluates evidence relevant to iPBHS across four inferential classes. Direct bioelectrochemical measurements connect viable electrode‐associated microorganisms to current or product formation [3, 4]. Mechanistic measurements resolve photon absorption, heat generation, interfacial charge transfer [30], ionic transport, and biomass‐specific conversion. Adjacent evidence establishes component function outside an integrated living reactor [16, 17, 31], whereas contextual evidence contributes transferable modelling or control principles [32, 33]. Comparisons use biological system, electrode architecture, illumination geometry, comparator design, spatial and temporal resolution, uncertainty, electron and carbon accounting, and operating scale. Quantitative synthesis is limited to compatible endpoints and denominators; otherwise, transfer functions, matched controls, and failure modes provide the basis for comparison. No complete integrated iPBHS prototype was identified in the reviewed literature; the term is therefore used as a testable reference architecture derived from the evidence hierarchy, not as an established technology class or a demonstrated unified mechanism.

## 2 Methodology

### 2.1 Review Scope and Evidence Identification

The evidence synthesis was conducted as a targeted, mechanism‐centered critical review designed to evaluate the scientific basis, integration requirements and experimentally testable boundaries of intelligent plasmonic bio‐holoelectrochemical systems. The literature domain encompassed microbial electrosynthesis and extracellular electron transfer, optically patterned and light‐responsive biofilms, plasmonic and photoelectrochemical interfaces, spatial electrochemical measurement, reactive transport, reduced‐order modelling, model‐predictive control, reactor scale‐up, and energy and life‐cycle accounting. Studies were considered relevant when they provided experimentally resolved mechanisms, quantitative electrochemical or biochemical performance data, appropriately defined comparators, spatial or temporal transfer functions, validated sensing or control strategies, durability information, or mass, charge, energy and carbon accounting applicable to living bioelectrochemical systems. Component‐level studies were retained where the underlying physical, electrochemical, biological, or computational principle was directly transferable to the proposed architecture. Cross‐domain studies were considered only when they established a relevant measurement method, control formulation, mechanistic discriminator, scaling constraint, or identifiable failure mode. This scope maintained emphasis on experimentally interpretable relationships while limiting extrapolation based solely on technological similarity.

### 2.2 Evidence Classification

Evidence was organized into four analytical categories according to its proximity to the integrated bioelectrochemical process: direct, mechanistic, adjacent, and contextual. Direct evidence comprised measurements linking viable electrode‐associated microorganisms to extracellular electron transfer, current generation, product formation, or spatially resolved electrochemical responses. Mechanistic evidence included studies capable of distinguishing optical, thermal, structural, electrochemical, transport, or biological contributions through controlled perturbations, orthogonal measurements, or matched comparators. Adjacent evidence described experimentally established component capabilities outside an integrated living reactor, including optical actuation, plasmonic sensing, spatial patterning, and process‐control technologies. Contextual evidence contributed transferable principles in modelling, parameter estimation, experimental design, statistical inference, reactor scale‐up, environmental accounting, or control theory. The classification was used to distinguish experimentally demonstrated system behavior from mechanistic inference and technology transfer, thereby preventing component‐level performance from being interpreted as evidence of integrated iPBHS functionality.

### 2.3 Comparative Evidence Synthesis

Comparative synthesis was conducted using common analytical dimensions across the different evidence categories. These dimensions included comparator fidelity, biological system, electrode architecture, illumination geometry, reactor scale, spatial and temporal resolution, normalization basis, state observability, parameter identifiability, measurement uncertainty, durability, and the closure of mass, charge, electron, energy, and carbon balances. Particular emphasis was placed on whether observed responses could be separated from competing explanations such as photothermal heating, changes in electrochemically active area, conductivity, optical attenuation, mass‐transfer variation, photocatalytic reactions, or biological stress. Evidence obtained under matched controls and independently resolved physical variables was assigned greater mechanistic weight than evidence based solely on changes in bulk current, product concentration or overall reactor performance. Differences among studies were interpreted in relation to experimental configuration, measurement basis and system boundaries rather than treated as equivalent estimates of a common response.

### 2.4 Quantitative Comparability and Uncertainty

Numerical results were compared directly only where organisms, operating conditions, reference states, normalization denominators, analytical endpoints and system boundaries were sufficiently compatible. Quantitative values derived from different reactor configurations, microbial communities, illumination conditions, or reporting bases were not pooled or combined into aggregate performance estimates. Where direct numerical comparison was not defensible, the evidence was synthesized through mechanistic transfer functions, matched‐control requirements, scale‐dependent failure modes, uncertainty sources, and the measurements required to resolve competing interpretations. Reported effect magnitudes were therefore used to characterize the range and context of experimentally observed responses rather than to generate multiplicative or pooled predictions for the integrated architecture. Measurement uncertainty, biological variability, model uncertainty, and parameter non‐identifiability were treated as distinct sources of uncertainty because they affect mechanistic interpretation and control performance differently.

### 2.5 Integration of Evidence into the iPBHS Framework

The final synthesis integrated the evidence according to the sequence by which optical actuation is translated into measurable bioelectrochemical performance. Evidence was first evaluated at the optical and material interface, followed by spatial electrochemical transfer, biological electron and carbon conversion, state estimation and feedback control, and finally durability, scale‐up, and system‐level accounting. Relationships were incorporated into the iPBHS framework only when the corresponding input, response, and major confounding variables could be defined quantitatively. Where integrated experimental evidence was unavailable, the relevant component capability was retained as an adjacent or contextual element rather than treated as demonstrated reactor‐level functionality. This approach enabled experimentally established processes, mechanistically supported relationships, transferable enabling technologies, and unresolved integration requirements to be represented within a single analytical framework without assigning equivalent evidential weight to fundamentally different types of studies.

## 3 Physical Architecture and Mechanistic Basis of iPBHS

The reference iPBHS configuration is a transparent flow‐through cathodic microbial‐electrosynthesis reactor in which optical actuation, electrode polarization, microbial conversion, sensing and control share closed mass, charge, energy and information boundaries. The optical subsystem comprises a narrow‐band LED or laser, a digital micromirror device or spatial light modulator, relay optics, and a transparent reactor window, consistent with established optogenetic patterning [16] and structured or holographic light‐delivery platforms [34, 35, 36]. Optical power, wavelength, projected pattern, and photon‐dose distribution are treated as calibrated actuator variables rather than nominal source settings, because optical propagation, attenuation [37], and registration [35, 36, 38] determine the field delivered to the bioelectrode.

**FIGURE 1:**
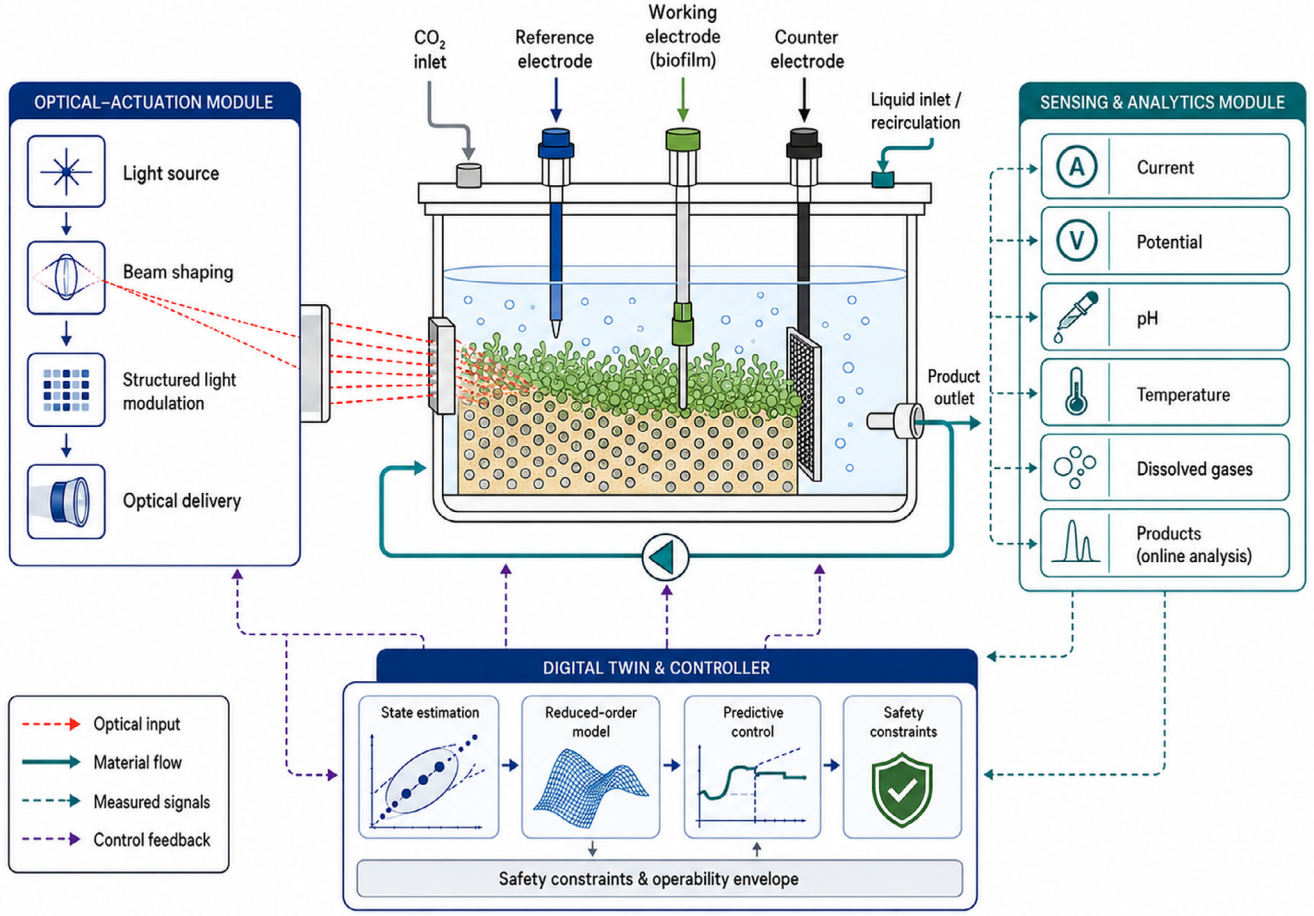
Coupled hardware, signal and control architecture of iPBHS. Author‐generated conceptual synthesis defining the reference hardware partitions and material, measurement and feedback pathways; no published experimental panel is reproduced.

The electrochemical subsystem comprises working, counter, and reference electrodes, gas and liquid delivery, recirculation, and product sampling. The measurement vector contains current, potential, impedance, pH, redox state, temperature, dissolved gases, biomass, and products [3, 4, 39], whereas the manipulated‐input vector contains electrode potential, photon flux, wavelength, spatial pattern, liquid flow, and CO_2_ delivery [32]. Structural identifiability and state observability therefore depend on calibrated actuator‐response transfer functions [32, 33], orthogonal controls and simultaneous mass, charge, energy and carbon balances, with uncertainty propagated across each measurement and state‐estimation layer [40, 41] (Figure 1).

Reproducible bench‐scale evaluation depends on an explicit design envelope rather than nominal equipment descriptions [5, 42]. The reported design variables include reactor volume, illuminated area, optical path length, electrode spacing, hydraulic residence time, gas and liquid flow, current density, photon flux, wavelength bandwidth, spatial pixel size [16], control interval, sensor sampling frequency [32], biofilm thickness [42, 43], and campaign duration, together with their calibration uncertainty. The relevant operating range is established from the transfer functions of the selected reactor and organism rather than imposed as a universal value, because optical attenuation, mass transfer [42, 43], and growth kinetics [3, 4] scale differently across geometries. Porous‐electrode modelling and scale‐up studies likewise identify flow distribution [43], gas retention [44], hydrodynamic non‐uniformity [45, 46] and transport path length [43, 46] as design variables that cannot be represented by electrode area alone.

Physical fluxes and latent reactor states follow different estimation pathways. Photon dose, electrical charge, gas and liquid flow, substrate consumption, and product formation can be calibrated directly, whereas biofilm activity, local transport resistance, and community state are reconstructed from sensor data and uncertainty‐aware models [32, 33]. A control‐induced improvement is identifiable where the actuator‐response relation remains stationary over the evaluation interval and when temperature, hydrodynamics, biomass, and analytical drift are independently resolved (Figure 1). Observer performance is therefore benchmarked against independently measured states or controlled perturbations; otherwise, improved tracking can arise from compensation of model bias rather than improved process knowledge [32, 40, 41]. Residual analysis and uncertainty calibration are used to distinguish sensor or model drift from biological non‐stationarity [47, 48].

### 3.1 Plasmonic Microbe Coupling: Optical, Thermal and Structural Discrimination

Nanostructured interfaces can increase extracellular electron transfer by changing conductivity, active area, roughness, and cell attachment [9, 10]. Gold nanoparticle modification of Saccharomyces cerevisiae produced a larger current response than conductive‐polymer modification [9]. Because illumination, action spectrum, local temperature, electrochemically active area, particle loading, and viability were not independently controlled, the experiment quantifies material‐enhanced current rather than resonant plasmonic modulation. The inferred mechanism is consequently non‐identifiable because the material modification changes several covariates simultaneously; wavelength‐resolved perturbation and a matched nonplasmonic control provide separation between optical excitation and passive electrode enhancement [11]. Recent cathode studies using nitrogen‐doped carbon dots [49], potential‐controlled electron‐transfer analysis [50], bimetallic Fe‐Mn oxides [51], NiFe‐layered oxides with carbon nanotubes [52] and CoNi‐CNT composites [53] further show that apparent current gains can arise from coupled changes in electron uptake, surface chemistry, hydrogen evolution and microbial enrichment. Mechanistic work in Geobacter further shows that multiple periplasmic cytochromes can inject electrons into OmcS nanowires through distinct extracellular electron‐transfer pathways [54].

**FIGURE 2:**
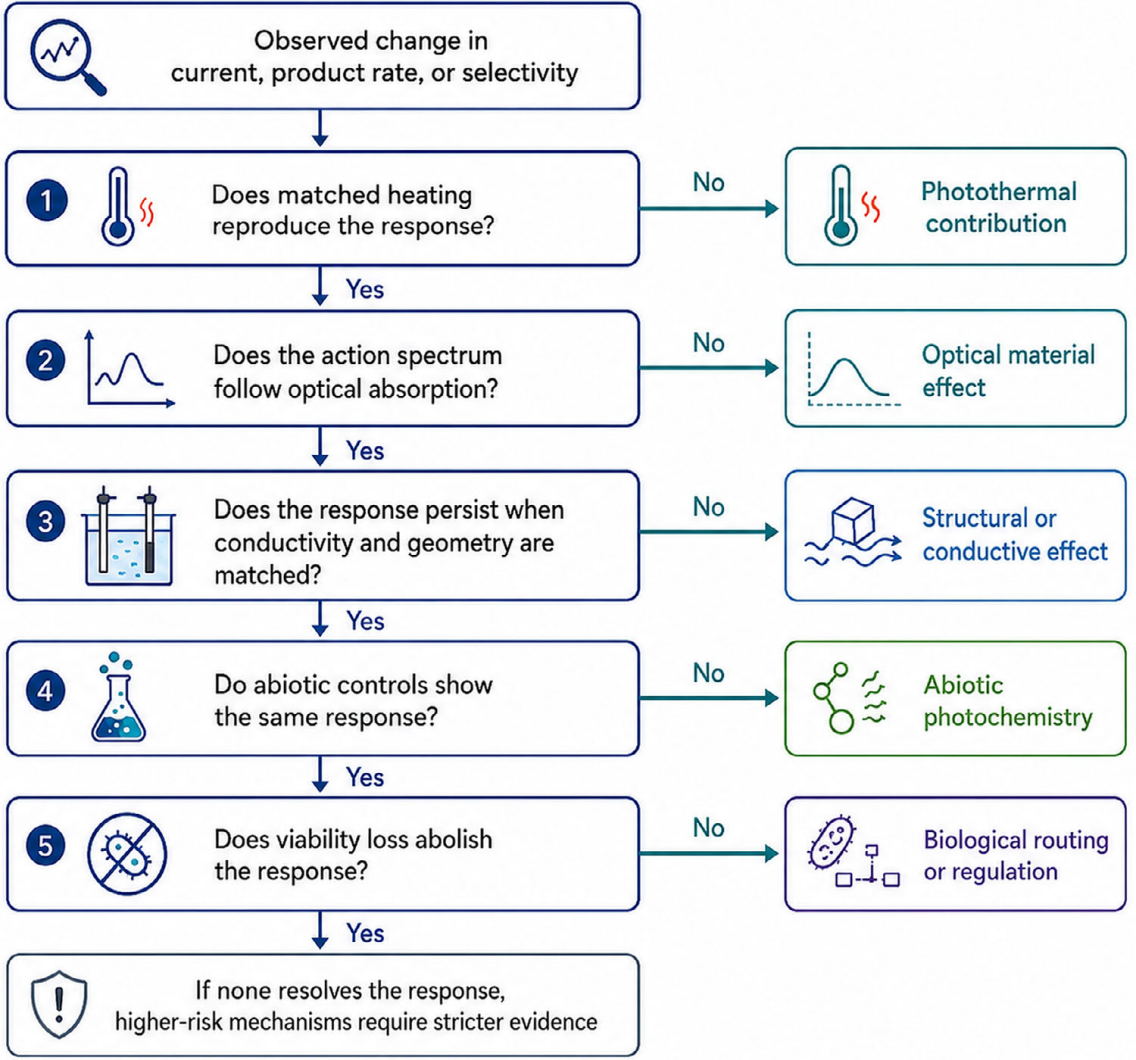
Hierarchical discrimination of illumination‐dependent bioelectrochemical mechanisms. Author‐generated decision pathway synthesizing the orthogonal controls required for mechanistic attribution; no published experimental panel is reproduced.

Plasmonic microbe coupling is identified through orthogonal optical, thermal, structural, and biological perturbations [11, 55]. Temperature‐equivalent heating quantifies photothermal contributions; action‐spectrum agreement tests wavelength specificity; conductivity‐, roughness‐, and area‐matched interfaces isolate morphology and charge‐transport effects; and abiotic and viability controls separate photocatalysis from metabolically dependent EET [9, 10, 11]. The resulting attribution sequence links an illumination perturbation to a specific interfacial mechanism rather than to an undifferentiated increase in current (Figure 2).

A spectral response alone is insufficient to identify plasmonically modulated EET. Resonant and off‐resonant illumination quantify wavelength dependence, external heating establishes thermal equivalence, and a nonplasmonic surface matched for conductivity, roughness, and electrochemically active area isolates the optical contribution [11, 12]. The final mechanistic assignment is determined by the set of alternative models that remain statistically compatible with the measured current, impedance, temperature, and viability trajectories. Spectral alignment is also evaluated against the absorption or scattering profile of the nanostructure, with response amplitude normalized to absorbed photon flux rather than incident irradiance [11, 12, 55]. This normalization prevents wavelength‐dependent optical losses from being misinterpreted as weaker interfacial coupling. Contemporary plasmon‐assisted electrochemistry and optoelectronics further separate photothermal heating [12, 56], hot‐carrier collection [57, 58], and wavelength‐intensity sensitivity [59] through calibrated thermal controls [12], fluence dependence [56, 58], and carrier‐dynamic measurements [57, 59], reinforcing the need for orthogonal optical and thermal attribution.

The grape‐like composite reported by Meng et al. increased bioelectrochemical performance through high surface area, conductive pathways, and microbial community selection [10]. Its porous morphology simultaneously changes electrochemically active area, conductivity, adsorption, biofilm loading, and local mass transport, preventing unique assignment of the current increase to one mechanism. Resonant plasmonic modulation instead is indicated by a wavelength‐dependent response that persists under conductivity‐, area‐, temperature‐, and dark‐matched controls. Procedures that distinguish photothermal from hot‐carrier effects provide the appropriate mechanistic design [11].

**FIGURE 3:**
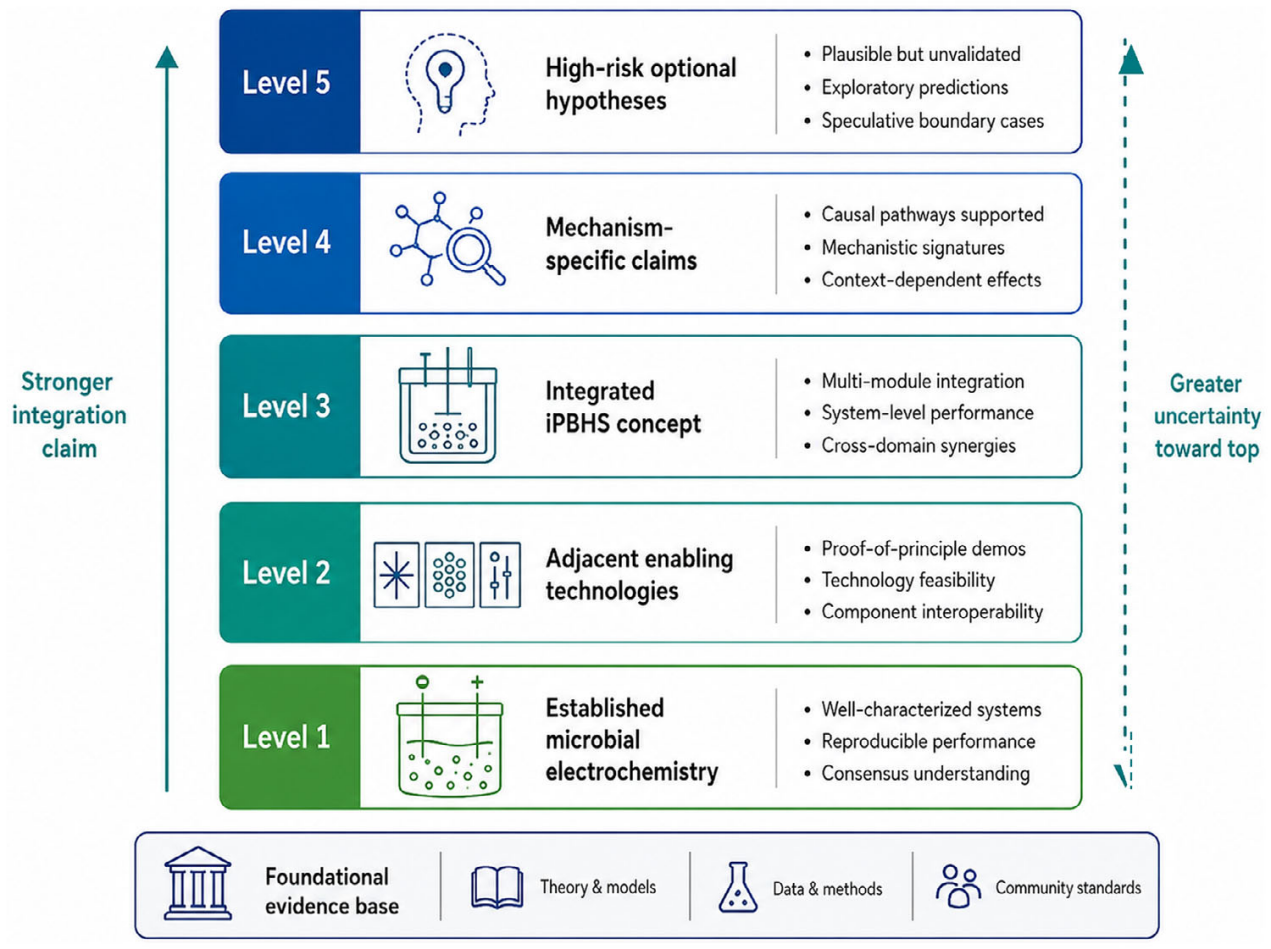
Evidence hierarchy for iPBHS integration. Author‐generated evidence hierarchy distinguishing established evidence, adjacent enabling technologies, integrated‐system claims, mechanism‐specific claims, and high‐uncertainty hypotheses.

The constituent technologies occupy distinct levels of experimental maturity. Established microbial EET [3] and adjacent photonic [11, 55], sensing [55], and control components [32, 33] provide the basis for integrated reactor testing, whereas plasmonically modulated EET and coherence‐sensitive catalysis depend on additional mechanistic observables (Figure 3). Measurement directness, comparator fidelity, uncertainty, and operating scale therefore determine the level of mechanistic resolution. Interpretation at a higher integration level remains defensible where lower‐level transfer functions remain measurable under the same operating conditions; otherwise, the integrated response cannot be assigned to a specific optical, electrochemical or biological process [11, 33, 55].

Direct plasmonic modulation of microbial EET has not yet been demonstrated under orthogonal optical, thermal, and structural controls. Optically addressable plasmonic sensors and actuators establish resonant control of nanostructures [55], whereas plasmonically modulated EET would require a reproducible change in biomass‐normalized EET under resonant illumination, attenuation of the response off resonance, equivalence with temperature‐matched heating, and preserved viability [11, 55]. A nonplasmonic electrode matched for conductivity, electrochemically active area, and roughness is the structural comparator. In the absence of this combined evidence, the experimentally resolved phenomenon should be described as nanomaterial‐enhanced EET rather than plasmonically modulated EET.

### 3.2 Holoelectrochemical Modulation: Spatial Transfer from Photon Field to Electrochemical Field

Spatial electrochemical control is relevant because electroactive biofilms develop gradients in redox state, potential, substrate availability, and community composition [60, 61]. Ion‐selective electrochemical modulation has generated microscale ionic environments with altered neural stimulation selectivity [62], demonstrating that local electrochemical fields can be manipulated independently of the bulk phase. Light‐patterned electroactive biofilms provide a closer precedent because geometry and conductivity were controlled on transparent electrodes [16]. The unresolved variable is the transfer function between a projected photon field and the local current‐density or overpotential field in an established submerged biofilm. Recent electroactive‐biofilm work directly resolves redox‐dependent spatial heterogeneity [60, 61] and cell‐surface electron‐transfer constraints [63, 64], showing that local electrochemical state and cytochrome organization can vary substantially within nominally uniform biofilms.

Structured‐light actuation encompasses amplitude‐patterned illumination generated by a digital micromirror device or spatial light modulator [16, 34], whereas holographic actuation additionally controls optical phase or wavefront structure [35, 36]. Within this framework, holoelectrochemical modulation denotes the broader transfer of a programmed photon field into a spatially resolved electrochemical response; amplitude‐only implementations are identified explicitly as structured illumination, and holographic terminology is reserved for phase‐ or interference‐controlled fields [34, 35, 36]. The transfer function is evaluated from co‐registered photon‐dose and electrochemical maps [65, 66, 67] acquired under equal integrated dose, with spatial frequency [34], phase [34], contrast [68], registration uncertainty [67], and thermal equivalence reported explicitly.

**FIGURE 4:**
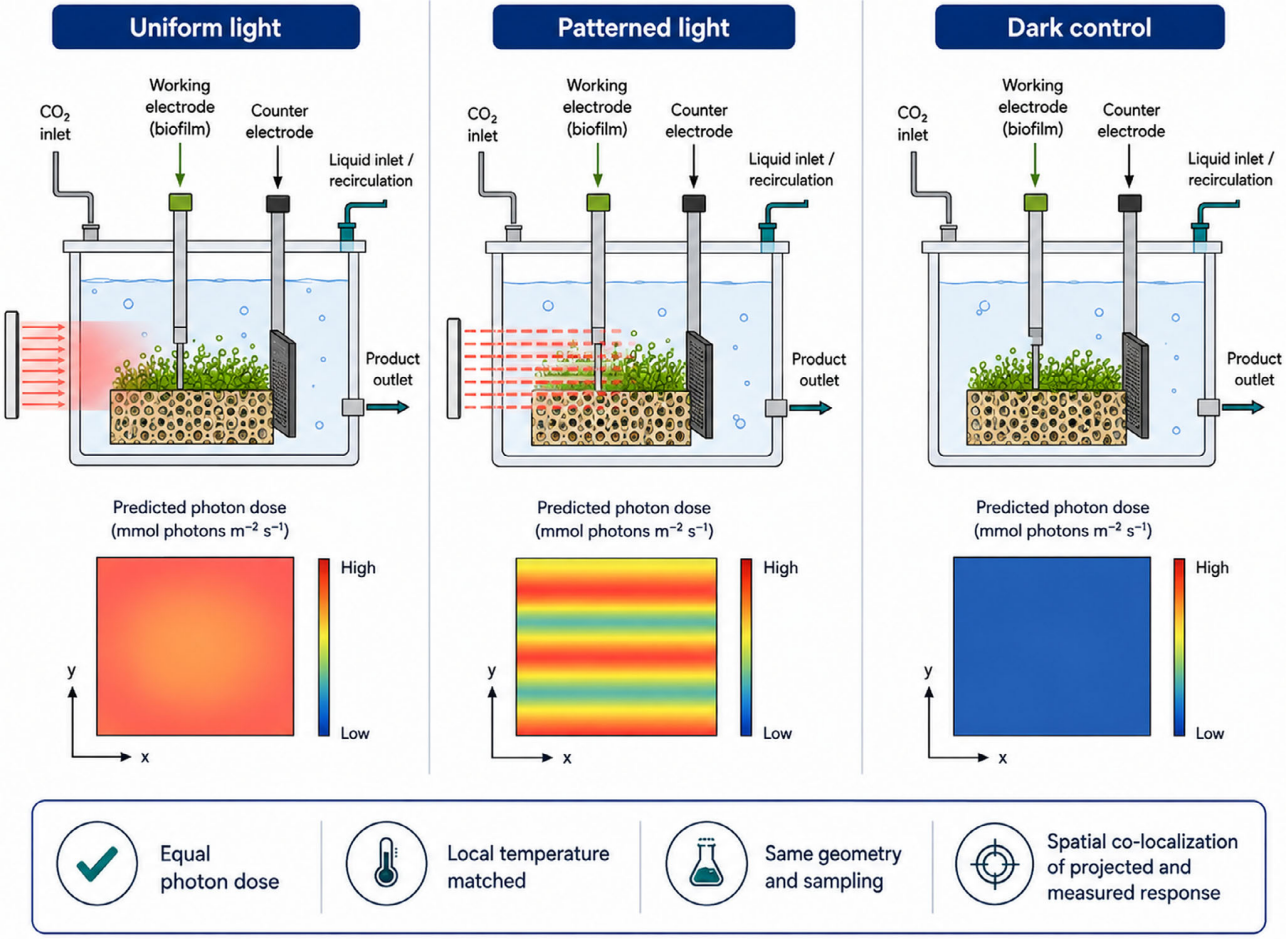
Equal‐photon‐dose framework for quantifying structured‐light electrochemical modulation. Author‐generated experimental comparison defining equal‐photon‐dose spatial validation; the reactor and field maps are schematic rather than experimental data.

Bulk production does not resolve spatial actuation because lateral conduction [63], diffusion, biofilm heterogeneity [60, 61, 64], and optical attenuation can erase an imposed pattern [16] while preserving the integrated output. Spatial transfer is therefore quantified from co‐registered photon‐dose and current‐density or potential maps acquired under equal integrated dose, temperature equivalence, and identical reactor geometry (Figure 4). Mechanistic resolution additionally depends on the modulation‐transfer function across spatial frequency, because a single correlation coefficient cannot distinguish true pattern transfer from coarse smoothing [34, 68]. Replicate maps [65, 66] and registration uncertainty [67, 68] determine whether spatial agreement exceeds that expected from background gradients. Micron‐scale redox mapping [65], scanning electrochemical microscopy [66], force‐coupled electrochemical imaging [67], quantitative spatial‐resolution analysis [68] and neutral‐atom surface imaging [69] provide complementary routes for co‐registering local chemistry, topography and electrochemical activity.

Holoelectrochemical modulation is an input‐output transfer property rather than a change in mean current [34]. Recovery of the imposed spatial pattern in the electrochemical field indicates local actuation, whereas loss of spatial frequency, phase, or contrast identifies attenuation [16], lateral charge redistribution [63], or convective‐diffusive transport as dominant limitations. Under the latter condition, illumination may enhance bulk bioelectrochemistry without providing spatial control. The transfer function is reported in amplitude and phase across relevant spatial scales, together with uncertainty from image registration, electrode segmentation, and local‐probe calibration [65, 66, 67]. These quantities distinguish genuine local control from apparent co‐localization produced by low‐pass transport or spatial averaging [68]. New biofilm studies also combine light‐directed patterning [70], nanostructured‐electrode electrochemistry [71], gene‐expression tracking [72], real‐time current diagnostics [73], in situ thickness monitoring [74], directional electrode architectures [75] and image‐based modelling [76] to resolve how local structure translates into electrical function.

### 3.3 Boundary of Plasmonic‐Field‐Assisted Biocatalysis

Illumination can alter interfacial reaction rates through local heating [11, 12, 56], hot‐carrier transfer [57, 58, 77], photochemical redox processes [77, 78], and changes in electrode polarization [12]. These mechanisms are distinguished through wavelength‐resolved kinetics [59, 77], temperature‐equivalent controls [11, 12], matched nonplasmonic interfaces [11], and quantitative carrier [57, 58, 77] or energy‐balance measurements [78]. Ultrafast hot‐carrier studies show that plasmon‐driven reaction pathways can require femtosecond‐resolved measurements for mechanistic attribution [77], while nanoscale energy‐balance analysis can quantify the relative contributions of hot carriers and photothermal heating [78]. Coherence‐sensitive catalysis remains outside the core iPBHS architecture because no reactor‐scale study has demonstrated controllable coherence‐dependent kinetics in a living bioelectrode; it is retained only as an optional spectroscopic boundary case requiring ultrafast or isotope‐sensitive observables after conventional photothermal and photochemical explanations have been excluded [20, 21].

### 3.4 Autonomous Flux Feedback and Digital‐Twin Control

Data‐driven control becomes technically interpretable through explicit definition of manipulated inputs, measured outputs, latent states, and constraints. AI methods have been applied to electrochemical sensing and bioelectrochemical‐system analysis [18, 39, 79], while data‐driven learning has been used for biocatalytic synthesis planning [80]. Reduced‐order machine‐learning models have also been embedded in model‐predictive bioreactor control and benchmarked against mechanistic and statistical alternatives [32]. Physics‐informed neural control has been applied to steer microbiome evolution toward higher polyhydroxyalkanoate production capacity [47]. These studies establish nonlinear prediction and constrained optimization in biological processes, but an iPBHS digital twin additionally combines joint estimation of optical field, interfacial electrochemistry, transport, and microbial state within the same reactor. Recent bioprocess‐control studies demonstrate that CFD‐based digital twins, model‐predictive control, and reinforcement‐learning controllers can be validated against independent process trajectories while retaining explicit operating constraints [40, 41, 81].

**FIGURE 5:**
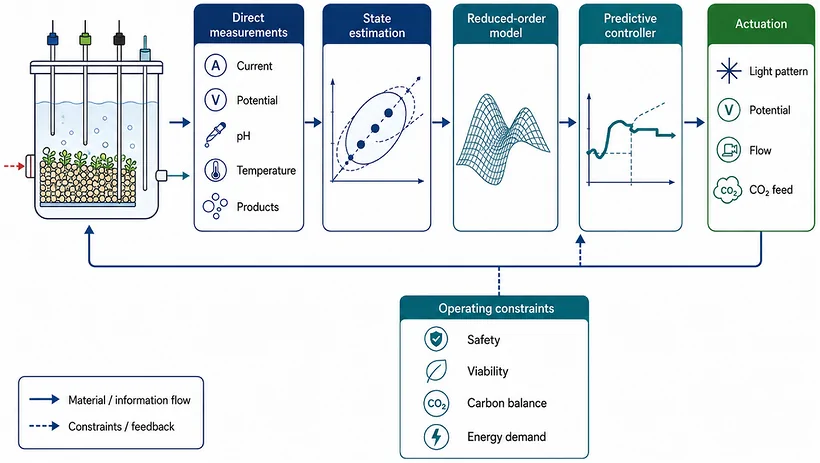
Bidirectionally synchronized digital‐twin control architecture. Author‐generated control schematic separating direct measurements, state estimation, reduced‐order prediction, constrained control, and actuation.

The digital twin is defined as a bidirectionally synchronized reactor model rather than a static soft sensor [33, 82]. Direct measurements update state and parameter estimates at each control interval; the reduced‐order model predicts candidate trajectories and uncertainty; the controller computes constrained actions; and prediction residuals trigger model recalibration, drift detection, or fallback operation [32, 40, 41]. The state vector includes current, electrode potential, charge‐transfer resistance, pH, temperature, biomass, viability, substrate, dissolved inorganic carbon, and product concentration, while manipulated variables include potential, photon flux, wavelength, spatial pattern, liquid flow, and CO_2_ feed. Digital‐twin performance is therefore assessed by synchronization error [40], held‐out trajectory prediction [41], parameter stability [32], uncertainty calibration [47], closed‐loop tracking [32, 41], and constraint compliance [47]. Additional work on economic nonlinear MPC [83], multiscale bioreactor models [84, 85], academic bioprocess digitalization [82], recurrent‐network MPC [86], and uncertainty‐aware control [87] shows that model synchronization, scale transfer, and constraint handling must be evaluated separately from nominal prediction accuracy (Figure 5).

### 3.5 Prior‐Art Integration Matrix

Existing platforms distribute the constituent functions across separate experimental systems. Photo‐assisted microbial electrosynthesis resolves light‐enhanced carbon conversion [30, 31], optogenetic biofilms resolve spatial biomass organization [16, 17], plasmonic photoelectrochemistry resolves wavelength‐dependent interfacial processes [11, 55], and model‐predictive bioreactors resolve nonlinear state estimation and constrained actuation [32, 47, 88]. iPBHS is an integrative reference architecture that combines co‐registered optical actuation and electrochemical sensing in a living bioelectrode while retaining viability, charge, carbon, and energy accounting. The relevant platforms are therefore compared by resolved capability, unresolved state variable, and the discriminating measurement that supports transfer to an integrated reactor (Table 1).

**TABLE 1:** Directly demonstrated capabilities, unresolved integration variables, and discriminating measurements across the principal evidence platforms.

Platform or evidence source	Directly demonstrated capability	Unresolved integration variable	Discriminating measurement	Refs.
Conventional microbial electrosynthesis	Electrode‐driven CO_2_ conversion and product formation	No spatial optical actuation	Electron recovery, carbon balance and product selectivity under calibrated potential	[3, 4]
Optogenetic electroactive biofilm	Light‐defined biomass geometry and electrical conduction	Pattern formation is not dynamic local overpotential control	Co‐registered photon‐dose and current‐density maps under equal dose	[16, 17]
Photo‐assisted microbial electrosynthesis	Illumination‐enhanced acetate production	Thermal, structural and spatial contributions remain coupled	Resonant/off‐resonant and temperature‐matched response with carbon/electron balances	[30, 31]
Plasmonic photoelectrochemistry	Wavelength‐dependent near‐field, hot‐carrier and photothermal effects in abiotic interfaces	Transfer to microbial EET is unresolved	Action spectrum, local temperature and matched nonplasmonic interface	[11, 55]
Spatial electrochemical/photonic actuation	Local ionic or electromagnetic fields can be generated and measured	Transport and fouling can erase the imposed pattern	Spatial transfer function in conductive, fouling electrolyte	[34, 62]
Digital‐twin and MPC bioreactor control	Reduced‐order prediction and constrained closed‐loop operation	No co‐registered optical, electrochemical and microbial state	Synchronization error, held‐out prediction, tracking and constraint compliance	[32, 33, 47]

The engineering value of iPBHS is evaluated against simpler alternatives under common denominators. Relevant comparators include uniform illumination [16], a conductivity‐ and area‐matched nonplasmonic electrode [11], a static high‐area electrode [89], segmented‐electrode actuation without structured light [16], fixed‐potential operation, a tuned PID controller, and conventional MPC without spatial optical inputs [32]. Incremental value [90, 91] is supported only where improved spatial selectivity, product distribution, or disturbance rejection persists after the additional optical power, sensing burden [5], calibration demand, maintenance requirements [5], and life‐cycle burdens [22, 92, 93] are included.

## 4 Reduced‐Order Multiphysics Model and Factorial Interaction Analysis

Online control depends on a model order that preserves the dominant optical, electrochemical, transport, and biological dynamics while remaining computationally compatible with the sampling interval [32]. The formulation is control‐oriented: it specifies state variables, conservation relations, disturbance terms, and validation structure, but predictive validity depends on parameter identification, independent calibration, and held‐out trajectory testing. Detailed electrodynamic and reactive‐transport simulations can constrain priors, whereas reduced‐order balances and data‐driven residual models support online estimation [33].

### 4.1 Coupled Optical, Electrochemical, Transport and Biological States

A control‐oriented model resolves the four state domains linking actuation to outcome: optical delivery, interfacial electrochemistry, mass transport, and biological conversion. The biological domain is non‐stationary because population composition and cross‐feeding can change while aggregate output remains similar. Multi‐metabolite cross‐feeding and intermediate‐responsive biosensors can stabilize population ratios and reduce toxic intermediate accumulation under a fixed production objective [94]. This evidence concerns compositional stabilization rather than dynamic reconfiguration under changing feed, photon distribution, or product target. A defined strain or low‐complexity consortium therefore minimizes ecological confounding during initial optical‐electrochemical system identification. Recent synthetic‐consortium studies emphasize ecological homeostasis [95], engineered habitats [96], division of labor and controlled cross‐feeding [97, 98] as design variables that can stabilize function while preserving measurable community interactions.

Online representation of community dynamics is limited by observability rather than model complexity. Automated community‐design approaches can identify robust interaction structures in silico [99], whereas multi‐metabolite cross‐feeding and intermediate‐responsive feedback demonstrate how designed interactions can stabilize population composition experimentally [94]. These approaches estimate or impose interaction structure under defined assumptions, but real‐time implementation depends on whether the corresponding states are observable from reactor sensors. Weakly identifiable interaction coefficients are therefore represented as probability distributions with propagated uncertainty rather than fixed parameters. Current guidance on synthetic microbial communities further stresses rigorous community definition [100], spatial organization [101], higher‐order interaction effects [102], and evolutionary stability [103], all of which become relevant when consortium state is treated as a controlled reactor variable.

Metabolic bypasses can overcome kinetic, thermodynamic, or redox restrictions [104], and mixture‐design methods can compose consortia from functional traits [105]. These approaches inform strain and consortium selection but do not resolve light‐induced pathway switching. Online inference is anchored to measurable uptake, production, and electron‐transfer rates, whereas pathway‐specific assignment of altered acetate selectivity is resolved through isotope tracing, targeted metabolomics, transcriptomics, or enzyme‐level measurements. Stoichiometric contribution, pathway reversibility, and uncertainty in flux attribution are reported for each inferred bypass so that model complexity is tied to an experimentally observable function rather than to network size alone. Bioelectrochemical evidence also shows that nonlinear relationships between active biomass and pollutant‐removal capacity can emerge from evolving interspecific interactions, supporting explicit treatment of community composition as a dynamic state rather than a fixed parameter [106].

Online regulation comprises state reconstruction, forward prediction, and constrained optimization. The observer estimates unmeasured biofilm and transport states from synchronized current, potential, pH, temperature, and product measurements; the forward model propagates those states under candidate optical and electrochemical inputs; and the optimizer minimizes tracking error and auxiliary energy while respecting viability and conservation constraints [32, 33, 47]. A separate randomized factorial model estimates optical‐interface‐control‐biological interactions [107, 108, 109], thereby distinguishing prediction accuracy, control efficacy, and cross‐module interaction.

Predictive accuracy, mechanistic identifiability, and control efficacy are orthogonal validation dimensions. Held‐out trajectory prediction quantifies model generalization [32, 40, 41]; matched physical perturbations resolve interfacial mechanism [11, 12]; closed‐loop experiments quantify tracking and constraint compliance [32, 47, 88]; and replicated factorial contrasts estimate departure from additivity [107, 108, 109]. Agreement across these dimensions strengthens interpretation, but no single dimension substitutes for the others. A control model can therefore be operationally useful without being mechanistically complete, whereas a mechanistically plausible model can remain unsuitable for real‐time optimization if its states are weakly observable or computationally intractable. These dimensions are reported separately.

### 4.2 Governing Equations and Identifiable Interaction Terms

The reduced‐order state representation couples four domains [32, 33, 43]. The optical balance maps incident irradiance and wavelength to absorbed photon flux and local heat generation. The electrochemical balance maps local overpotential, charge‐transfer resistance, and active area to interfacial current. Convection‐diffusion‐reaction equations describe CO_2_, ionic species, substrates, and products in the bulk liquid and biofilm, while biomass‐specific kinetics map chemical and electrical state to EET and product formation. Community drifts [106], fouling, and sensor bias [48] enter as stochastic disturbances, allowing state uncertainty to propagate into control decisions [32, 47].

Digital twins for electro‐physical, chemical, and photonic processes demonstrate progression from monitoring to prescriptive control [33]. Reliability is highest where geometry, material properties and boundary conditions are well characterized. Living biofilms add state‐dependent kinetics, structural change and stochastic community responses, and consequently depend on mechanistic constraints, residual correction, parameter regularization and explicit prediction intervals [40, 84, 85]. A controller that increases mean output while increasing constraint violations, temperature excursions, or product variance provides no net control benefit [32, 47, 88].

Equations (1)–(6) close the reduced‐order model through wavelength‐dependent photon attenuation, species conservation, biomass‐normalized EET kinetics, nonlinear state estimation, constrained optimization, and factorial interaction. The formulation defines the measurable outputs, latent states, uncertain parameters, disturbance terms, and penalty functions used for online estimation and independent validation. Each balance is written on a common control volume and time base so that optical absorption, species transport, current generation, and product formation remain dimensionally consistent. Parameter priors and disturbance covariances are estimated independently of validation trajectories to avoid optimistic model fit.

The optical state begins with conversion of incident spectral irradiance into a local absorbed‐photon source term. Equation (1) includes photon energy, effective absorption density, and depth‐dependent attenuation so that the result has units of absorbed photons per unit volume and time; the attenuation and absorption terms are obtained from calibrated optical measurements. Plasmonic studies that explicitly quantify photothermal heating and optical response provide the experimental basis for separating absorbed optical power from thermal effects [11, 12].

(1) $$q_{\mathrm{abs}}\;\left(x,y,z,\lambda ,t\right)=\frac{I_{0}\left(x,y,\lambda ,t\right)\;\lambda }{\mathrm{hc}}\;\;\alpha_{\mathrm{eff}}\left(\lambda ,\xi \right)\exp\left[-\mu_{\mathrm{eff}}\left(\lambda ,t\right)z\right]$$

Transport of dissolved species is represented here by a Nernst–Planck–reaction balance. Equation (2) includes convection, molecular diffusion, and electric‐field‐driven migration, while the electroneutrality condition constrains ionic concentrations in the bulk electrolyte; interfacial flux boundary conditions couple species consumption or production to measured current. This formulation is consistent with the need to resolve ionic transport, reaction, and current distribution in microbial‐electrosynthesis and porous‐electrode models [43, 110, 111].

(2) $$\frac{\partial C_{i}}{\partial t}+\nabla \cdot \;\left(uC_{i}-D_{i}\nabla C_{i}-z_{i}u_{i}FC_{i}\nabla \phi \right)=\nu_{i}\;r_{i}\left(C,E,T,B\right),\;\sum_{i}z_{i}\;C_{i}=0$$

Biomass‐normalized extracellular electron transfer is represented here by a multiplicative kinetic relation in Equation (3). The biological terms are anchored to established microbial electrosynthesis [3, 4], while substrate availability, overpotential, temperature, and absorbed photon flux enter as separable response functions so that illumination is not double‐counted as both an additive current source and a temperature‐dependent kinetic effect [11, 12]. Direct protein‐level work in Geobacter biofilms further shows that extracellular electron‐transfer proteins contribute measurably to ferric‐mineral reduction, providing an experimental basis for separating biological electron‐transfer capacity from purely interfacial conductivity [112].

(3) $$j_{\mathrm{EET}}=j_{0}\;B\;f_{S}\left(C_{S}\right)\;f_{\eta }\left(\eta \right)\;f_{T}\left(T\right)\;f_{\Phi }\left(q_{\mathrm{abs}}\right)$$

The digital‐twin state estimator is written in discrete time because measurements and control actions occur at finite sampling intervals. Equation (4) separates process dynamics, manipulated inputs, uncertain parameters, process disturbances, and measurement noise, thereby defining the quantities used for filtering [32], moving‐horizon estimation [40, 41], and residual‐based drift detection [47].

(4) $$x_{k+1}=\;f\left(x_{k},u_{k},\theta \right)+w_{k},\;y_{k}=\;h\left(x_{k},u_{k}\right)+v_{k}$$

Constraint‐aware control uses an objective defined over a prediction horizon and normalized so that tracking, resource use, and safety penalties are commensurable [32, 47, 88]. Equation (5) therefore minimizes dimensionless tracking error and resource use while penalizing temperature, viability, and actuator‐limit violations at each predicted step [41, 47, 88].

(5) $$J\;=\sum_{j=0}^{N_{p}-1}\left[w_{p}{\tilde{e}}_{p,k+j}^{2}+w_{E}{\tilde{E}}_{\mathrm{aux},k+j}+w_{T}\Phi_{T}+w_{v}\Phi_{\mathrm{viability}}+w_{c}\Phi_{\mathrm{constraints}}\right]\;$$

Cross‐module interaction is estimated with the complete four‐factor regression in Equation (6) [107, 108, 109]. The model includes interface, illumination pattern, control mode, and biological state as coded factors, together with all two‐, three‐, and four‐way interactions; biological replicate, electrode batch, and experimental day are represented separately as blocking or random effects [107, 108, 109].

(6) $$Y\;=\beta_{0}+\sum_{i\;=1}^{4}\beta_{i}X_{i}+\sum_{i<j}\beta_{\mathrm{ij}}X_{i}X_{j}+\sum_{i<j<k}\beta_{\mathrm{ijk}}X_{i}X_{j}X_{k}+\beta_{1234}X_{1}X_{2}X_{3}X_{4}+\varepsilon$$

In Equations (1)–(6), q_abs is the volumetric absorbed‐photon rate; I_0_ is incident spectral irradiance; α_eff and μ_eff are effective absorption and attenuation coefficients; C_i_, D_i_, z_i_ and u_i_ are species concentration, diffusivity, charge and ionic mobility; φ is electric potential; j_EET is biomass‐normalized extracellular electron‐transfer current; x and y are state and measurement vectors; and X_1_–X_4_ encode interface, illumination, control mode and biological state. All variables are assigned SI or explicitly reactor‐normalized units, and interaction coefficients are reported with covariance, confidence intervals, and model diagnostics consistent with design‐of‐experiments [107, 108, 109] and equivalence‐analysis principles [113].

The spatial model uses measured inlet concentrations and flow as Dirichlet or convective inlet conditions, convective outflow at the reactor outlet, zero normal flux at impermeable walls, and current‐linked molar flux at the bioelectrode. Initial liquid concentrations, biomass inventory, and electrode state are obtained from independent measurements before each run. Optical and transport parameters are estimated from calibration experiments, kinetic parameters from non‐optical electrochemical data, and disturbance covariances from replicate residuals. A finite‐volume or method‐of‐lines discretization with implicit time integration is suitable for the stiff transport‐reaction subsystem, while state estimation and nonlinear MPC operate on the reduced model [32, 33, 43].

Calibration and validation are separated. Parameter estimation uses designated calibration trajectories spanning the intended input domain [32, 40]; structural and practical identifiability are evaluated before controller tuning [108]; and predictive performance is tested on held‐out disturbances, illumination patterns, and biological replicates [41, 109]. Sensitivity analysis reports which outputs constrain each parameter, and uncertainty intervals are propagated into control actions and factorial estimates [47, 113]. The formulation is therefore evaluated as a control‐oriented model whose predictive validity is determined experimentally rather than inferred from equation structure alone.

Component‐level studies report markedly different response magnitudes [30, 31]. An optical light‐management layer in photo‐assisted microbial electrosynthesis increased acetate production by up to 242% and 543% in two configurations, with coulombic efficiencies of 70% and 81% and a reported solar‐to‐acetate efficiency of 3.20% [31]. A distinct engineered photoelectrochemical interface increased acetate production by 55.6% [30]. These values were obtained with different organisms, interfaces, illumination fields, baselines, and normalization methods; they therefore cannot be combined multiplicatively. More generally, life‐cycle [22, 93, 114], techno‐economic [91, 92], and bioelectrochemical sustainability analyses [5] show that improvement in a focal performance metric need not translate into net system benefit. In iPBHS, increases in acetate rate can therefore be offset by auxiliary electricity, optical heating, toxicity, carbon loss, or reduced long‐term stability. More recent microbial‐electrosynthesis studies span Fe@NC hybrid cathodes [115], catalyst screening [116], carbon‐negative wastewater integration [117], cathodic‐pH control [118], hypersaline operation [119], moderate‐salinity gas‐diffusion biocathodes [120], silica‐nanoparticle enhancement [121], and hydrogen‐mediated mixed cultures [122], demonstrating that reactor chemistry and operating matrix strongly condition the attainable response.

**FIGURE 6:**
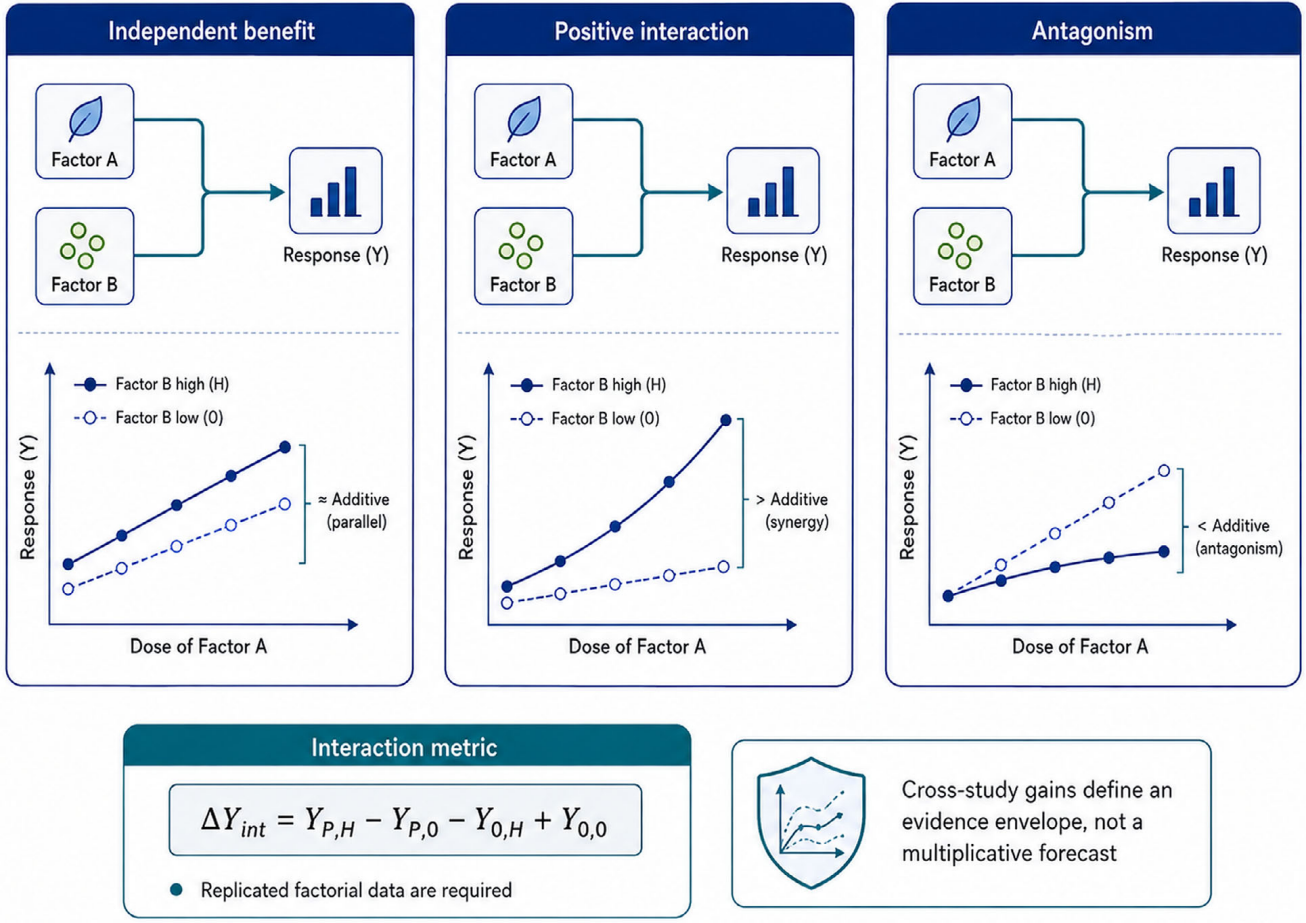
Factorial interpretation of additive effects, positive interaction, and antagonism. Author‐generated conceptual illustration of factorial interaction outcomes; plotted trends are schematic and are not pooled experimental data.

Cross‐study heterogeneity precludes retrospective factorial estimation [30, 31]. The reported acetate increases were obtained with different organisms, electrode chemistries, illumination protocols, baselines, durations, and normalization methods; treating them as cells of one factorial design would violate experimental‐unit and independence assumptions [107, 108, 109]. Published values therefore describe the range of component‐level response magnitudes, whereas cross‐module interaction is estimated within a single biological system using common denominators, synchronized sampling, and replicated factor combinations (Figure 6). Contemporary syntheses and experiments additionally show that organism choice [123], gas‐mediated electron delivery [124], medium‐chain‐product formation [125, 126], data‐driven optimization [127], electrode‐associated enrichment [128] and electron‐supply mode [129] can shift productivity and selectivity through different mechanisms, which further argues against pooling heterogeneous component gains.

A retrospective numerical ablation that pools published sub‐module effect sizes is likewise not treated as evidence of cross‐module synergy because the underlying studies [30, 31] do not share experimental units, biological states, denominators, or covariance structure [107, 108, 109]. Instead, ablation is defined prospectively within one calibrated iPBHS dataset: individual coupling terms are set to zero, or specific actuator channels are held constant, and the resulting changes in held‐out prediction error [32, 33], factorial interaction estimates [107, 108, 109], and constraint violations [32, 33] are compared with the full model. This preserves experimental independence and tests whether each optical, interfacial, biological, or control coupling contributes identifiable explanatory or control value.

### 4.3 Cross‐Study Comparability and Within‐System Factorial Design

Within‐system interaction analysis uses a randomized, blocked 2^4^ factorial design with four binary factors: plasmonic vs. matched nonplasmonic interface, patterned vs. uniform equal‐dose illumination, closed‐loop vs. fixed operation, and live vs. abiotic or killed‐biomass condition [107, 108, 109]. The full model estimates main effects, six two‐factor interactions, four three‐factor interactions, and the four‐way interaction, with biological replicate, electrode batch, and experimental day represented as blocking or random effects [107, 108, 109]. If resources do not support the complete design, a pre‐specified fractional factorial design preserves the interactions central to mechanistic attribution and reports its alias structure [107].

Pairwise interaction patterns provide an interpretable projection of the complete four‐factor regression (Figure 6), while Equation (6) retains all specified main and higher‐order effects. Pairwise plots are interpreted after higher‐order terms and blocking factors have been evaluated, because an apparent two‐factor interaction can change sign or magnitude when biological state or control mode is omitted [107, 108, 109]. Interaction estimates are reported with confidence intervals, model diagnostics, and sensitivity to the normalization denominator [107, 108, 109].

An SLM or DMD defines the illumination field, while phase‐change [130] and liquid‐crystal environments [131] can tune resonant optical behavior. Electrochromic systems provide a complementary materials precedent in which electrochemical state produces reversible changes in optical response [132, 133]. For two binary factors, the interaction term is the difference‐in‐differences between the combined condition and the corresponding main effects [107]. Positive interaction is supported when the interaction estimate exceeds zero, and its confidence interval excludes the null value; antagonism produces a negative estimate through attenuation, heating, toxicity, mass‐transfer limitation, or controller delay. Effect sizes from independent studies are excluded because they do not share a biological system, covariance structure, or normalization denominator. Candidate components are consequently compared by transfer function, matched control, dominant failure mode, and scale dependence (Table 2).

**TABLE 2:** Principal iPBHS components, matched comparators and scale‐dependent failure modes.

Component	Proposed function and principal variable	Matched comparator	Dominant failure or scale issue	Refs.
Plasmonic nanostructure	Optically addressable EET interface; resonance, absorbed photon flux and current	Nonplasmonic surface matched for area, conductivity and roughness	Heating, fouling, corrosion and ion release	[11, 55]
Transparent bioelectrode	Spatial electrochemical readout; active area and optical transmission	Opaque or unpatterned electrode with matched geometry	Optical loss, delamination and biofilm‐induced drift	[16, 62]
DMD or SLM	Programmed photon field; dose, wavelength and spatial frequency	Uniform illumination at equal integrated dose	Pattern distortion, attenuation and alignment drift	[16, 34]
Electrochemical and optical sensors	State estimation; calibration error, response time and cross‐sensitivity	Independent analytical reference and redundant channel	Biofouling, drift and reconstruction artefacts	[33, 38, 39]
Defined electroactive catalyst	Electron and carbon conversion; biomass, viability and product flux	Abiotic, killed‐biomass and simplified‐community controls	Community succession, toxicity and changing stoichiometry	[3, 4, 94]
Predictive controller	Tracking and disturbance rejection; horizon, uncertainty and constraints	Fixed policy, PID and MPC without spatial optical input	Model bias, latency and out‐of‐distribution operation	[32, 47, 88]
Gas/liquid delivery	CO_2_ supply and transport; flow, residence time and mass‐transfer coefficient	Matched hydrodynamics and gas transfer across optical conditions	Bubbles, pH gradients, optical attenuation and scale dependence	[42, 110, 111]

## 5 Mechanism‐Resolved Validation Metrics and Uncertainty Analysis

Mechanistic resolution depends on synchronized measurement of optical input [11], electrode state [62], biological activity [16], and product formation [3, 4]. A cathodic CO_2_‐to‐acetate reactor provides electron‐ and carbon‐balance observables [3, 4], whereas a transparent anodic biofilm provides spatial EET and interfacial‐conductivity observables [16, 70]. Introducing multiple contaminants, mixed products, or distributed coordination before these transfer functions are identified increases parameter correlation and structural non‐identifiability. The initial validation domain is therefore defined by the smallest reactor configuration that retains the optical‐electrochemical‐biological coupling of interest.

Statistical identifiability depends on independent biological replication, a pre‐specified primary endpoint, and sample‐size justification based on expected variance and effect magnitude [107, 108, 109]. Electrode potential, photon dose, hydrodynamics, gas delivery, biomass loading, pH, temperature, and sampling time are controlled within experimentally defined ranges. Thermal and viability equivalence are evaluated using calibrated measurement uncertainty and pre‐specified equivalence or non‐inferiority margins [113]. Randomization and blocking account for electrode batch, inoculum, reactor position, and day‐to‐day analytical variation [107, 108, 109], while repeated measurements are modelled as correlated observations rather than independent replicates. This structure prevents technical replication from inflating effective sample size.

The minimum viable iPBHS experiment is therefore a bench‐scale transparent reactor containing a defined electroactive biofilm and an optically addressable working electrode, operated first without adaptive feedback. The decisive interface comparison comprises dark, resonant, off‐resonant, and temperature‐equivalent illumination on a plasmonic surface together with a nonplasmonic surface matched for conductivity, electrochemically active area, and roughness [11, 12]. The primary endpoint is biomass‐normalized EET, supported by impedance, local temperature, and viability measurements; progression requires a replicated interface‐by‐illumination interaction [107, 108, 109] whose uncertainty excludes the pre‐specified null or equivalence margin [113]. Only after this criterion is met is patterned illumination introduced under equal photon dose, with co‐registered local current or potential maps [65, 66, 67] used to test spatial transfer [16, 62, 68]. Carbon‐product selectivity and closed‐loop control are evaluated only after interfacial and spatial transfer functions remain identifiable, preventing a system‐level gain from substituting for failure at a lower mechanistic level.

### 5.1 Interface Attribution: Separating Optical, Thermal and Material Effects

Bioelectrochemical systems exhibit a persistent trade‐off among energy density, durability, and validation quality [5]. Conductive and catalytic cathodes, including amine‐functionalized metal‐organic frameworks, can improve CO_2_ reduction and acetate production [134], but these responses remain material effects unless illumination is treated as an independent experimental factor [107]. Interface attribution compares a plasmonic electrode with a nonplasmonic electrode matched for conductivity, electrochemically active area, and roughness across resonant, off‐resonant, dark, and temperature‐equivalent conditions under common hydrodynamics [11, 12]. Area‐ and biomass‐normalized current, impedance‐derived charge‐transfer resistance, action spectrum, local temperature, and viability provide the discriminating readouts. Recent optogenetic and spatial‐biofilm studies demonstrate that illumination can be used to alter electroactive‐biofilm response [135], cell‐cell adhesion [136], community patterning [137], surface morphology [138], and single‐cell expression trajectories [139], providing direct experimental precedents for spatially programmed biological states.

Multi‐criterion economic model‐predictive control demonstrates how productivity, substrate use, and operational constraints can be optimized within a common bioprocess formulation [88]. For interface characterization, adaptive control is disabled so that feedback compensation cannot obscure the physical origin of the measured response. Plasmonic modulation is quantified from a replicated illumination‐by‐interface interaction in biomass‐normalized EET [107, 108, 109], spectral agreement with the optical resonance [11], thermal equivalence [12, 113], and preserved viability [113]. Absence of this interaction indicates nanostructured bioelectrochemistry without resolved plasmonic modulation.

Measurement requirements expand with system complexity [5, 42]. Interfacial photoresponse is resolved through electrochemical, optical, and thermal readouts; spatial actuation additionally uses co‐registered field maps; closed‐loop performance uses synchronized state and actuator trajectories; carbon routing is assessed through elemental and electron‐equivalent balances [140, 141]; and sustainability is assessed through a life‐cycle inventory linked to a functional unit [22, 92]. These measurement layers prevent an increase in one aggregate endpoint from obscuring failure in another subsystem [5]. For each layer, calibration error is propagated into endpoint uncertainty so that gains in an aggregate response cannot be interpreted independently of the measurement resolution supporting that response [113].

Quantitative criteria depend on instrument precision, biological variance, spatial resolution, and the selected equivalence, non‐inferiority, or superiority margin [113]. Universal percentages are not transferable across reactor geometries and organisms because detection limits and biologically relevant effect sizes vary with electrode area, biomass loading, optical path length, and analytical method [42]. Numerical thresholds are interpretable when reported with their denominator, confidence interval, calibration model and uncertainty‐propagation method [113]. Equivalence margins are therefore justified from assay performance and the minimum biologically important difference, with sensitivity analysis used to determine whether conclusions change across defensible margin choices [113].

### 5.2 Spatial Actuation Under Equal‐Photon‐Dose Conditions

Microbial electrosynthesis converts CO_2_ to acetate, but productivity and selectivity depend on cathode chemistry [49, 50, 115], microbial physiology [110, 111], and operating conditions [119]. Spatial actuation is quantified where patterned illumination produces a local electrochemical field under an unchanged integrated photon dose. Local current or potential can be resolved with scanning electrochemical methods [65, 66, 67] or spatially resolved electrode readouts [68]. The primary response is spatial correspondence between the imposed photon field and the measured electrochemical field, with bulk production retained as a secondary endpoint.

Scale‐up studies identify mass transport [43, 44], pH gradients, electrode area [42], separator losses [45], and reactor hydrodynamics [46] as persistent constraints. During spatial attribution, these variables are held equivalent across illumination conditions. Spatial holoelectrochemical modulation is quantified by a spatial correlation coefficient with confidence interval, a target‐to‐background response ratio, temperature equivalence defined from calibrated sensor uncertainty, and viability equivalence defined from the assay‐specific non‐inferiority margin [113]. Spatial resolution and optical transfer‐function bandwidth are reported because apparent correlation can be inflated by smoothing or undersampling [34, 68].

### 5.3 Closed‐Loop Control, Carbon Routing and Complex Matrices

Closed‐loop efficacy is quantified under identical sensing, actuation, and constraint conditions. Fixed operation, a tuned PID or rule‐based controller, and model‐predictive control [32, 40, 41] receive the same measurements and operate within the same potential, photon‐dose, temperature, flow, and viability limits [47, 88]. The primary metrics are set‐point tracking error [81, 86], disturbance‐rejection time [87], constraint violations [83, 88], and energy‐normalized product formation [83, 88]. A control advantage is indicated by improved tracking or selectivity without a compensating increase in auxiliary energy, temperature excursions, biological instability, or carbon imbalance. In complex matrices, recent bioelectrochemical work on simultaneous chloramphenicol and polyvinyl‐chloride transformation illustrates why contaminant removal should be evaluated with compound‐specific conversion endpoints rather than inferred from aggregate current alone [23].

Complex wastewater is an informative robustness matrix only after the optical‐electrochemical transfer functions have been resolved. Bioelectrochemical treatment has recovered energy while degrading industrial coffee wastewater [142], but variable chromophores, suspended solids, ionic strength, organic matter, and optical scavengers alter photon transport, electrode kinetics, and analytical recovery simultaneously. Multi‐contaminant performance is therefore evaluated through mass balances for parent compounds and transformation products, toxicity assays, adsorption controls, and energy‐normalized removal [23, 142].

**FIGURE 7:**
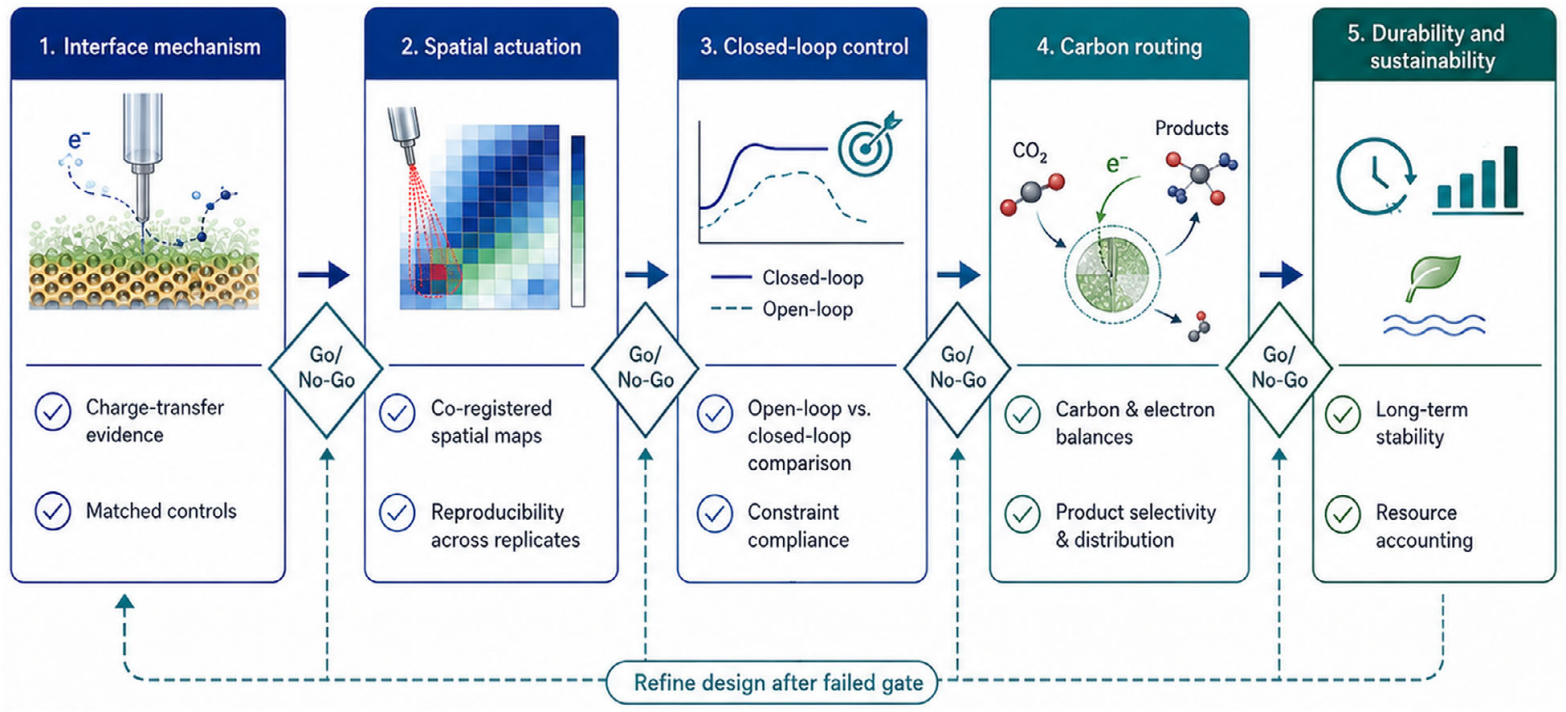
Stage‐gated validation from interfacial mechanism to durability and sustainability. Author‐generated stage‐gated validation roadmap; decision margins are experiment‐ and uncertainty‐dependent rather than universal thresholds.

Integrated performance remains interpretable only while lower‐level transfer functions remain identifiable [11, 32, 33]. The validation hierarchy therefore couples interface attribution and spatial actuation to closed‐loop control, electron and elemental balances [140, 141], durability [5, 42] and resource accounting [22, 92]; evaluation at a higher integration level retains the controls and diagnostics established at the preceding level (Figure 7). This nesting permits failures to be localized to an actuator, interface, transport process, state estimator, or accounting boundary instead of being absorbed into an undifferentiated system‐level metric.

The hierarchical sequence prevents bulk acetate production, contaminant removal, or predictive accuracy from masking loss of optical transfer, interfacial electrochemical resolution, or biological stability. Failure at a given level localizes the dominant deficiency to optical delivery, electrode architecture, sensing, state estimation, biological stability, or system accounting, thereby preserving causal interpretability of the integrated response. Loss of calibration in a preceding transfer function, uncertainty beyond the specified decision margin, or conservation residuals indicating an unmeasured flux define stopping conditions for further integration. Operationally, failure is assigned when the interface‐by‐illumination effect [107, 108, 109] is not distinguishable from the pre‐specified null or equivalence margin [113], spatial correspondence does not exceed registration and measurement uncertainty, closed‐loop operation does not outperform its matched controller comparator under the same constraints, or charge, elemental, or carbon balances fail their uncertainty‐derived closure criteria [140, 141, 143].

## 6 Failure Propagation and System‐Level Accounting

Failure propagation arises from coupling among actuator, process, and measurement states. Optical attenuation changes the delivered photon field [37]; surface fouling changes resonance, charge‐transfer resistance, and active area [5, 42]; community drift changes kinetic parameters and product stoichiometry [106]; and sensor [39, 48] or model bias [32, 47] changes state estimates and controller action. System performance therefore includes calibration stability [32], parameter identifiability [48], fault detection [47], uncertainty propagation [48], and fallback control [32, 42] in addition to mean current or product rate [5].

### 6.1 Optical Attenuation, Field Distortion and Reactor Scale

Digital plasmonic holography and nanoparticle color holograms demonstrate precise wavefront engineering in dry or optically controlled environments [35, 36]. Plasmonic structures integrated into photonic waveguides further demonstrate confined optical‐mode routing [37]. In a turbid ionic reactor, biofilm growth, gas bubbles, suspended solids, and refractive‐index gradients distort phase and amplitude, while metallic and electrolyte losses reduce penetration depth. The relevant scale is set by the measured optical transfer function and attenuation length, not by geometric reactor area alone.

Label‐free surface‐plasmon‐resonance holographic microscopy can monitor refractive‐index and interfacial changes [38]. In iPBHS, a reference optical channel estimates pattern position, contrast, and intensity at the electrode independently of the biological response [35, 36]. When spatial contrast falls below the calibrated detection limit, transitioning to uniform illumination or dark electrochemical operation limits model compensation from concealing loss of optical actuation. Repeated independent optical calibration accounts for the fact that refractive‐index shifts and fouling can change both signal amplitude and spatial phase [37]. Reference‐channel residuals become a direct input to state uncertainty rather than a post hoc quality flag.

Scale‐up performance depends on independent detection and containment of optical, interfacial, biological, and control drift [38, 42]. Calibration channels, analytical standards, local fallback policies, and safe operating envelopes remain active at each deployment level so that node‐level mass, charge, and carbon balances are not obscured by supervisory optimization [24, 38]. Supervisory coordination remains bounded by validated local operating envelopes, while calibration residuals or mass‐balance errors beyond specified limits interrupt cross‐node aggregation [24]. This architecture limits propagation of undetected bias.

Aggregate output cannot localize a failed module because optical attenuation, electrode fouling, biological drift, and sensor bias can produce similar declines in current or product rate. Fault isolation therefore combines reference optical channels [38], electrochemical diagnostics [39, 79], analytical standards, community‐state measurements, and out‐of‐distribution detection [48] before the controller changes the operating point. This separation prevents compensation in one module from concealing degradation in another. Fault‐signature verification uses injected optical, electrochemical, and sensor perturbations so that the diagnostic classifier is challenged against known failure modes before autonomous recovery is enabled [32, 33, 48].

### 6.2 Biological Stability, Phototoxicity and Model Reliability

Phototoxicity, ion release, and biofilm stress are more immediate sources of biological non‐stationarity than coherence‐sensitive effects. Optical dose [11], local temperature [12, 56], reactive oxygen species, material stability [5], viability, membrane integrity, and community composition are therefore monitored together during prolonged illumination. Quantum‐coherence studies provide specialized spectroscopic concepts, but they do not establish a controllable reactor‐scale mechanism and do not constitute part of the core architecture [20, 21].

Microbial drift is a direct source of model non‐stationarity. Community succession and evolving interspecific interactions can alter electron partitioning, pollutant transformation, and apparent kinetic parameters [106]. AI approaches have been evaluated for bioelectrochemical optimization [18, 79], but nominal predictive accuracy is insufficient when the operating state shifts beyond the training domain; autonomous use therefore requires drift detection, uncertainty calibration, out‐of‐distribution recognition, and a verified fallback policy [48, 144]. A state estimator that cannot detect a changed biological state has limited suitability for closed‐loop actuation even when its training error is low.

### 6.3 Energy, Carbon and Remediation Accounting

Supervisory optimization operates above the reactor conservation layer [22, 92, 145] and cannot replace the mass, charge, and energy balances governing physical performance [140, 141, 143]. The operational energy inventory includes electrode power, optical power [22, 92, 145], pumping, gas compression [145], mixing, sensing, computation, and downstream separation [146]. These terms are integrated over the same temporal and spatial boundary as exported energy or product credit, using time‐resolved measurements rather than nominal equipment ratings [22, 92, 145]. System‐level evaluation should also include process economics and life‐cycle burdens [90, 91, 93]. Complementary reactor‐scale electrochemical studies further show that mass‐transfer selectivity and scale‐dependent transport can change performance as electrode architecture and reactor dimensions increase [147, 148].

Net electrical performance is evaluated from the complete operational energy balance in Equation (7), integrated over the same temporal and spatial boundary used for product formation and exported energy [22, 92, 145]. Net electricity generation corresponds to a positive value after electrode polarization, optical delivery [22, 92, 145], pumping, gas handling, mixing [145], sensing, computation, and product separation [146] are included. Cathodic microbial electrosynthesis is otherwise classified as electricity‐driven carbon conversion, and environmental comparison is conducted through a life‐cycle inventory linked to a declared functional unit, allocation procedure, and conventional comparator [22, 92].

Net electrical performance is defined on the same control volume and time interval as product formation. Equation (7) subtracts electrode polarization, optical delivery [22, 92, 145], pumping, gas handling, mixing [145], sensing, computation, and separation energy [146] from any exported electrical energy, preventing gross product value from being interpreted as net electricity generation.

(7) $$\begin{array}{cc} E_{\mathrm{net}} & =E_{\mathrm{export}}\;-\left(E_{\mathrm{electrode}}+E_{\mathrm{optical}}+E_{\mathrm{pumping}}+E_{\mathrm{gas}}+E_{\mathrm{mixing}}\right.\\ & \;+\left.E_{\mathrm{sensing}}+E_{\mathrm{computation}}+E_{\mathrm{separation}}\right) \end{array}$$

Carbon accounting is based on a molar balance across CO_2_, bicarbonate, organic feed, products, biomass, dissolved inorganic carbon, residual substrate and off‐gas. Carbon recovery is calculated with Equation (8) on a common sampling interval and control volume. Recovery between 90% and 110% has been used as a broad diagnostic range in reaction‐engineering and continuous‐culture studies, but even balances within 95%–105% can generate substantial error in calculated conversion or selectivity [140, 141, 143]. The acceptable closure interval is therefore derived from uncertainty in flow, concentration, gas composition, and biomass measurements rather than imposed as a universal threshold.

Carbon recovery includes changes in reactor inventory during transient operation. Equation (8) therefore combines carbon leaving in products, liquid, and gas [22, 92] with the measured change in dissolved, suspended, biofilm, and adsorbed carbon inventory [140, 141, 143] before comparison with total carbon input.

(8) $$R_{C}=\frac{\sum n_{C,\mathrm{out}}+\Delta n_{C,\mathrm{inventory}}}{\sum n_{C,\mathrm{in}}}\;\times 100\%$$

Pathway‐specific carbon partitioning is additionally resolved through carbon‐normalized selectivity, electron‐equivalent recovery, and isotope tracing when competing pathways cannot be separated stoichiometrically. The inventory term in Equation (8) includes dissolved inorganic carbon, residual substrate, suspended biomass, biofilm biomass, adsorbed carbon, and gas‐phase holdup over the common sampling interval. A 90%–110% recovery window can be used as a broad diagnostic check, but the acceptance interval is derived from uncertainty in flow, concentration, gas composition, and biomass measurements rather than treated as a universal threshold [140, 141, 143]. Net‐negative performance remains a life‐cycle property defined by a functional unit, system boundary, comparator, allocation method, and uncertainty analysis.

Biological validation, optical addressability, spatial readout, control maturity, and accounting depth provide common comparison axes for microbial electrochemical, photo‐assisted, patterned‐biofilm, plasmonic, and digital‐twin platforms (Table 3). The comparison identifies which coupled transport, electrochemical, biological and control functions have been demonstrated within each platform and which remain separated across systems. Using the same axes avoids ranking platforms by a single favorable endpoint and exposes trade‐offs among mechanistic resolution, operational maturity, spatial observability and accounting completeness.

**TABLE 3:** Comparative biological, optical, control and accounting maturity of iPBHS‐related platforms.

Platform	Biological conversion evidence	Optical/spatial capability	Control and accounting maturity	Refs.
Conventional MFC or MES	Direct EET, current generation or CO_2_‐to‐product conversion	No programmed spatial optical actuation	Moderate reactor maturity; carbon and energy accounting variable	[3, 4, 5, 42]
Photo‐assisted MES	Direct light‐enhanced microbial product formation	Bulk illumination; limited spatial resolution	Bench‐scale; thermal and auxiliary‐energy attribution incomplete	[30, 31]
Patterned electroactive biofilm	Direct link between light‐defined geometry and conductance	Millimeter‐scale spatial patterning	No dynamic product‐routing or feedback control	[16, 17]
Plasmonic photoelectrochemistry	Primarily abiotic reaction evidence	Strong spectral and near‐field control	High mechanistic resolution; limited biological durability data	[11, 55]
Digital‐twin bioreactor control	Bioprocess state prediction and closed‐loop control	No spatial optical bioelectrode	Strong control methodology; no microbial electrochemical carbon balance	[32, 33, 47, 88]
iPBHS reference architecture	Prospective integration of living bioelectrode, spatial optics and constrained control	Co‐registered photon and electrochemical fields	Requires integrated validation of transfer functions, durability and life‐cycle performance	Original synthesis

The related platforms are complementary rather than interchangeable. Conventional microbial electrochemical systems provide comparatively mature evidence for biological function [3, 4, 5] and scale‐up [42] but lack spatial optical actuation. Photo‐assisted systems demonstrate light‐enhanced carbon conversion with limited spatial resolution [30, 31]; patterned biofilms provide geometric control without dynamic electrochemical routing [16, 17]; plasmonic photoelectrochemistry resolves optical mechanisms primarily in abiotic interfaces [11, 55]; and digital‐twin bioreactors provide predictive control without a co‐registered optical bioelectrode [32, 33, 47]. iPBHS is differentiated by simultaneous registration of these functions within one measurement, control, and conservation architecture.

Evaluation of interface attribution, spatial actuation, feedback control, carbon routing, remediation, durability, and life‐cycle performance is organized around stage‐specific endpoints. Each integration stage is therefore defined by a primary endpoint, orthogonal controls, associated readouts, a quantitative decision criterion, and a mechanistic diagnosis (Table 4). The criteria are linked to measurement uncertainty and failure diagnosis, and progression remains interpretable while the preceding transfer function remains identifiable under the expanded operating domain.

**TABLE 4:** Stage‐specific endpoints, controls and quantitative interpretations for iPBHS evaluation.

Validation stage	Primary endpoint	Essential controls and readouts	Quantitative interpretation	Refs.
Plasmonic interface	Biomass‐normalized EET under illumination	Dark, off‐resonance, temperature‐equivalent, and matched nonplasmonic controls; current, EIS, temperature, and viability	Illumination‐by‐interface interaction with confidence interval, spectral agreement and equivalence within assay‐derived margins	[9, 10, 11, 55, 113]
Spatial actuation	Correspondence between photon‐dose and electrochemical fields	Uniform equal‐dose, dark and scrambled pattern; co‐registered dose, current/potential and temperature maps	Spatial transfer function, target/background ratio and registration uncertainty across spatial frequency	[16, 34, 62]
Closed‐loop control	Set‐point tracking or product‐selectivity control	Fixed policy, PID and MPC without spatial input; tracking error, violations, energy and viability	Improved tracking or disturbance rejection without increased constraint violations or auxiliary energy	[32, 47, 88]
Carbon routing	Carbon‐normalized product distribution	Common potential and photon dose; products, gas flow, DIC, biomass and current	Statistically resolved selectivity with carbon and electron‐equivalent balance closure including inventory change	[3, 4, 22, 30, 31, 92, 140, 141, 143]
Remediation robustness	Removal and detoxification of a named contaminant	Abiotic, adsorption, dark and conventional BES controls; parent compound, products, toxicity and energy	Improved removal with complete transformation‐product accounting and no increase in measured toxicity	[142]
Durability	Stable calibrated operation	Reference optical channel and periodic analytical standards; fouling, corrosion and community state	Performance and calibration remain within pre‐specified limits over dominant growth and fouling time constants	[5, 38, 42]
Life‐cycle performance	Net greenhouse‐gas balance for a declared functional unit	Complete energy/material inventory, conventional comparator, allocation and sensitivity cases	Net balance below zero across the stated system boundary and uncertainty analysis	[22, 92]

Common denominators and explicit diagnostic outcomes preserve interpretability across integration levels. A negative interaction estimate is interpreted through the pre‐specified factorial model [107, 108, 109], while incomplete elemental or carbon balances identify an accounting deficiency [140, 141, 143]. Net‐negative terminology is restricted to life‐cycle greenhouse‐gas balances below zero across the stated functional unit, system boundary, allocation method, and uncertainty analysis [22, 92, 93]. This boundary prevents product credits, avoided emissions, or partial carbon recovery from being combined across incompatible functional units or system boundaries.

## 7 Extended Integration Architectures

Once node‐level optical, electrochemical, and biological transfer functions are calibrated, replicated reactors can be coordinated through supervisory control without transferring safety‐critical functions away from the node. Local sensing, elemental balances and fallback control remain active during network‐level optimization. Distributed operation is therefore assessed against a single‐reactor baseline using throughput, optical‐path uniformity, resilience, energy demand, maintenance, latency and fault‐containment metrics. The supervisory layer optimizes allocation of feed, light and electrical resources but does not override local interlocks or calibration checks [24]. Communication loss therefore degrades performance to validated node‐level operation rather than producing an uncontrolled global state [25].

### 7.1 Distributed Reactor Networks and Supervisory Control

Industrial wireless‐control research identifies communication reliability, latency, and safety as design variables [24], while bio‐cyber interface studies identify parameter‐profiling and security risks [25]. These constraints are independent of reactor chemistry. Distributed control therefore retains node‐level safety functions, mass and charge balances, and fallback operation independently of supervisory allocation of flow, light, and electrical resources. Communication delays and packet loss are treated as bounded disturbances, and controller stability is assessed under worst‐case latency and node isolation. Cybersecurity monitoring is separated from biochemical state estimation so that anomalous commands cannot be mistaken for biological drift.

### 7.2 Programmable Consortia and Functional Complexity

Synthetic microbial consortia can be programmed through ecological interactions [149], and automated niche adjustment can stabilize natural and engineered cocultures [150]. These methods regulate population structure and resource exchange but do not resolve real‐time optical reprogramming of consortium topology [94, 99]. Additional community complexity is technically justified where each member contributes a measurable reaction step, electron flux, or carbon flux that cannot be supplied by a simpler system [104]. Stoichiometric contribution, stability under perturbation, and recoverability after member loss are demonstrated for each strain [105]. Omics‐only evidence is insufficient when the associated function cannot be linked to electron recovery, carbon flux, or product formation.

### 7.3 Learning, Adaptation and Biological Drift

Adaptive control distinguishes model updating from biological drift. Parameter adaptation is justified where prediction residuals, process constraints and independent biological measurements remain mutually consistent; otherwise, sensor failure, contamination or community succession can be absorbed incorrectly into the updated model [48, 106, 144]. Online drift detection, out‐of‐distribution recognition and fallback operation are therefore integral to state adaptation in bioelectrochemical control [48, 79]. Explainable real‐time bioprocess monitoring further demonstrates that prediction quality should be accompanied by interpretable diagnostics and robustness testing before model updates are allowed to influence autonomous operation [48].

**FIGURE 8:**
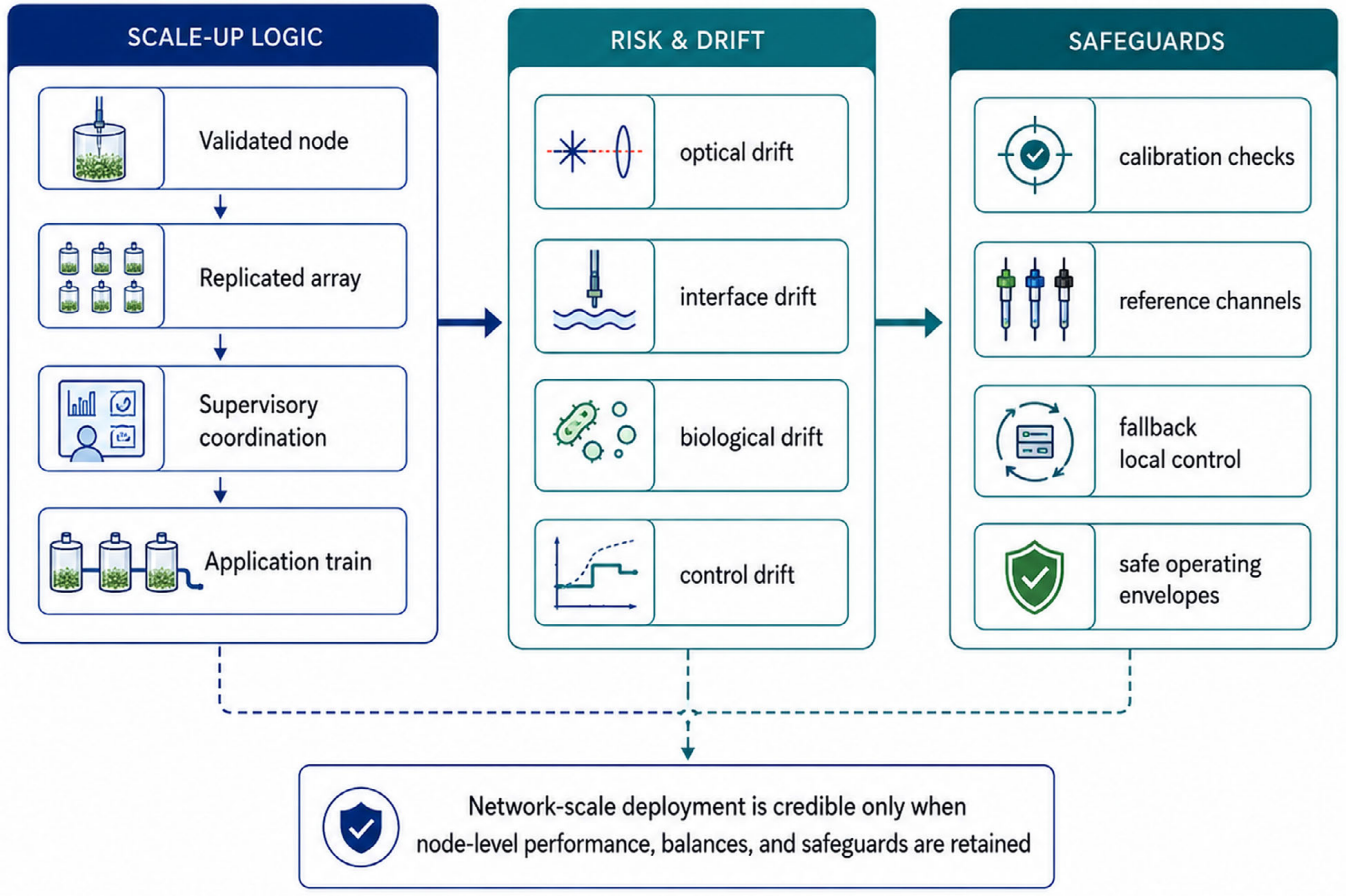
Scale‐up architecture, drift modes and safeguards for iPBHS deployment. Author‐generated scale‐up and fault‐containment architecture; the network arrangement is a testable engineering synthesis and does not imply an existing deployment.

Scale‐up shifts the dominant uncertainty from component function to inter‐node reproducibility, calibration stability, maintenance, and fault containment [42]. Replicated arrays and supervisory coordination retain reference channels, analytical standards, local fallback control, and safe operating envelopes at every deployment level, enabling drift to be localized before it propagates through the network (Figure 8) [24]. Cross‐node statistics separate between‐node variability from within‐node temporal drift, and reference channels are analyzed with the same sampling frequency as production signals. This permits early isolation of calibration loss before it propagates through supervisory optimization [25].

Distributed scaling trades shorter optical paths and fault isolation against duplicated sensors, communication latency, calibration burden, maintenance demand, and cyber‐physical risk [24, 25]. Network deployment provides a net engineering advantage where gains in throughput, resilience, or optical uniformity exceed these additional energy and operational costs relative to a simpler reactor architecture. Credible deployment assessment reports node‐level variance, communication availability, maintenance intervals, spare‐sensor requirements, and the energy cost of redundancy rather than extrapolating linearly from one reactor.

## 8 Conclusion and Outlook

iPBHS brings together experimentally established advances in microbial extracellular electron transfer, CO_2_‐to‐product electrosynthesis, structured‐light projection, optogenetic biofilm patterning, plasmonic photoelectrochemistry, electrochemical sensing, and model‐predictive control, but their integration remains an unresolved multiphysics and control challenge. Mechanistic validation requires wavelength‐dependent EET that persists after thermal and structural matching, spatial correspondence between projected photon fields and electrochemical responses, improved closed‐loop performance under viability and energy constraints, and simultaneous electron‐equivalent and carbon closure; increases in bulk productivity alone cannot establish these mechanisms. The immediate research priority is therefore rigorous integration rather than further accumulation of isolated component‐level gains. Actionable milestones are to: experimentally separate plasmonic, photothermal, structural, and biological effects using orthogonal controls; quantify spatial optical‐to‐electrochemical transfer with co‐registered measurements; validate state estimation and predictive control against independent reactor measurements; demonstrate charge, carbon, and energy closure under sustained operation; and establish stability across biofilm growth, fouling, calibration drift, and increasingly complex matrices. Translation beyond laboratory systems will additionally require modular reactor designs that preserve optical contrast and electrochemical reproducibility while controlling sensing burden, maintenance, communication latency and cyber‐physical risk. Progress will therefore depend on coordinated collaborations spanning microbial electrochemistry, photonics, materials science, process systems engineering and environmental assessment. Achieving these milestones would establish whether structured optical actuation delivers reproducible advantages over conventional electrode engineering and feedback control and define the engineering basis for credible scale‐up.

## Author Contributions

**Timoth Mkilima**: conceptualization, methodology, investigation, evidence synthesis, visualization, writing – original draft, writing – review and editing, and project administration.

## Funding

This work received no external funding.

## Conflicts of Interest

The author declares no conflicts of interest.

## Data Availability

Not applicable. This review did not generate or analyze any new datasets.
